# Combined Microscopic and Endoscopic Surgery for Pineal Region Meningiomas Using the Occipital-Parietal Transtentorial Approach

**DOI:** 10.3389/fonc.2022.828361

**Published:** 2022-02-04

**Authors:** Yu Ding, Liang Sun, Yukun Hu, Weiwei Zhai, Liexiang Zhang, Zhengquan Yu, Jiang Wu, Gang Chen

**Affiliations:** ^1^ Department of Neurosurgery, The First Affiliated Hospital of Soochow University, Suzhou, China; ^2^ Department of Neurosurgery, The Affiliated Suqian Hospital of Xuzhou Medical University, Suqian, China

**Keywords:** meningiomas, pineal, endoscope, trans-bitentorial approach, falcotentorial junction

## Abstract

**Objective:**

Pineal region meningiomas are deeply located and adjacent to critical neurovascular structures, making them one of the most challenging areas to access. The authors presented a combined microscopic and endoscopic surgery and investigated its value in resecting pineal region meningiomas.

**Methods:**

Twelve patients with pineal region meningiomas from February 2017 to December 2020 were retrospectively reviewed. All patients underwent combined microscopic and endoscopic surgery using the occipital-parietal transtentorial approach. Perioperative clinical, surgical, and radiographic data were collected.

**Results:**

The endoscope provided a wider view and increased visualization of residual tumors. All tumors were completely resected, and none of the patients died. Total resection was believed to have been achieved in four patients, but the residual tumor was detected after endoscopic exploration and was completely resected with an endoscope. Only one patient had transient visual field deficits. No recurrence was observed during follow-up.

**Conclusions:**

Combined microscopic-endoscopic surgery for pineal region meningiomas eliminates microscopic blind spots, thus compensating for the shortcomings of the traditional occipital transtentorial approach. It is a promising technique for minimally invasive maximal resection of pineal region meningiomas.

## Introduction

Meningiomas of the pineal region are rare, accounting for approximately 8% of tumors in this region (1, 2). According to the tumor origin, pineal region meningiomas are classified into two main subtypes: falcotentorial and velum interpositum meningiomas. Falcotentorial meningiomas originate from the junction of the dural folds between the falx cerebri and tentorium. In contrast, velum interpositum meningiomas arise from the double-layered pia of the velum interpositum and have no direct dural attachment (3–6).Most pineal region meningiomas grow slowly, occupying the pineal region and compressing surrounding structures, such as the pineal gland, midbrain, deep venous system, and the third ventricle. Due to the deep location and close relationship with critical neurovascular structures, radical resection of meningiomas in the pineal region remains a significant challenge.

Numerous surgical approaches have been advocated for the resection of meningiomas in the pineal region. The most commonly used approaches are occipital transtentorial and supracerebellar infratentorial approaches. However, both approaches have disadvantages. Visualization of the surgical field without blind spots is changing using these approaches because the tumor is obscured by the dural structures and deep venous system (7–9). Previous studies have reported various modifications to overcome these disadvantages, including the occipital bitranstentorial/falcine approach, bilateral occipital craniotomy, and simultaneous combined supratentorial/infratentorial approaches (10–12). These approaches provide a wider exposure but are associated with significant risks, such as venous infarction and cortical blindness.

The concept of using endoscopy to resect pineal lesions is not novel. However, most endoscopic approaches reported previously were through the infratentorial supracerebellar corridor to access the pineal region (13, 14). This study presents a combined microscopic and endoscopic surgery using the occipital-parietal transtentorial approach for the radical resection of pineal region meningiomas.

## Methods

### Patients

During this retrospective study, 12 patients at the First Affiliated Hospital of Soochow University underwent surgery to resect pineal region meningiomas from February 2017 to December 2020. All patients underwent combined microscopic and endoscopic surgery using the occipital-parietal transtentorial approach. Perioperative clinical, surgical, and radiographic data were collected.

There were four female and eight male patients, ranging in age from 39 to 71 (mean age, 54.3) years. The patients’ clinical course varied from 2 weeks to 4 months (median, 2.1 months). This study was approved by the local ethics committee(S2018087), and informed consent was obtained from the patients. Patient profiles are summarized in **Table 1**.

**Table 1:** Clinical characteristics of 12 patients undergoing the combined microscopic and endoscopic surgery for resection of pineal region meningiomas.

Case no.	Age (yrs), sex	Tumor size (mm)	Presenting symptoms	Hydrocephalus	EOR	Surgical complications	Dural attachment	Histological subtype	Post-op mRS score
1	42, M	25	Incidental	N/A	GTR	None	Tentorium	Fibrous	0
2	71, M	57	HA, vomiting, blurred vision	Yes, transient EVD post-op, resolved afterwards	GTR	Transient VFD	Tentorium/falx	Meningothelial	2
3	67, F	52	HA, blurred vision	Yes, transient EVD post-op, resolved afterwards	GTR	None	Tentorium/falx	Meningothelial	1
4	39, F	38	HA, vomiting	N/A	GTR	None	Falx	Transitional	0
5	49, F	37	HA, dizziness	N/A	GTR	None	Tentorium	Fibrous	0
6	39, M	41	HA	N/A	GTR	None	Tentorium/falx	Fibrous	1
7	57, M	43	HA, memory issues, imbalance	Yes, transient EVD post-op, resolved afterwards	GTR	None	Tentorium/falx	Psammomatous	1
8	52, F	26	Incidental	N/A	GTR	None	Velum interpositum	Meningothelial	0
9	45, M	29	Dizziness, fatigue	N/A	GTR	None	Tentorium	Meningothelial	0
10	65, M	34	Dizziness, memory issues	Yes, relieved after surgery	GTR	None	Tentorium	Transitional	0
11	58, M	31	Incidental	N/A	GTR	None	Tentorium/falx	Angioblastic	0
12	67, M	38	HA, dizziness	Yes, relieved after surgery	GTR	None	Tentorium/falx	Meningothelial	1

EVD, external ventricular drainage; HA, headache; N/A, no hydrocephalus; GTR, gross total resection; VFD, visual field defect.

### Combined Microscopic and Endoscopic Video Monitor System

**Figure 1:**
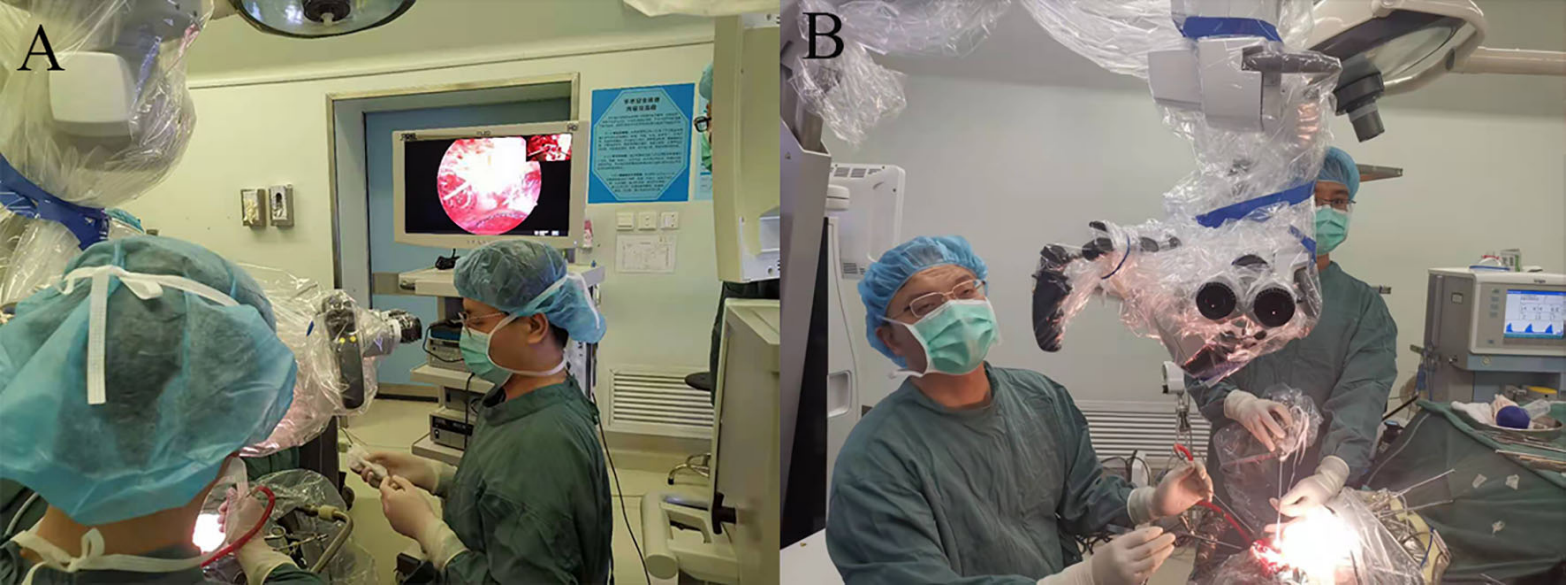
**(A)** Setup of the microscope, endoscope, and image-merged screen in the operating room. The introduction and movement of the endoscope are well supervised under microscopic view. The endoscopic view is displayed in full screen, and the microscopic view is shown in the upper right corner. **(B)** The position of the main surgeon and the assistant. The assistant holds the endoscope while the main surgeon performs bimanual dissection.

We used an endoscope (Karl Storz, Germany) and a microscope (Pentero; Carl Zeiss, Germany) in our integrated operating room. Both endoscopic and microscopic views were simultaneously displayed on a high-definition screen (picture-in-picture, **Figure 1A**) visible to the entire surgical team, including the surgeon, assistant surgeon, and assisting nurses.

### Neuroimaging

All patients underwent preoperative computed tomography and magnetic resonance imaging (MRI). Tumor size, peri-tumor edema, tumor shape, and hydrocephalus were assessed. In addition, magnetic resonance venography was performed before surgical intervention for multimodal fusion to evaluate the patency of the deep veins and to understand the location of the tumor in relation to the deep veins. The extent of resection (EOR) represents the percentage of the preoperative tumor volume resected (preoperative volume – postoperative volume)/preoperative volume. Tumor volume was measured on T1-weighted gadolinium-enhanced magnetic resonance images using iPlan Cranial software (BrainLab, Germany). Gross total resection (GTR) was defined as the tumor that was confirmed to be completely resected by the surgeon with no residual tumor detected on postoperative contrast MRI (EOR = 100%). Gross total resection included Simpson grades I-III. Near-total resection was defined as the resection of more than 90% of the neoplasm (90 ≤ EOR <100%), and subtotal resection was defined as the resection of less than 90% of the neoplasm (EOR < 90%).

### Follow−Up and Outcome Evaluation

Follow-up imaging was performed immediately (<3 days), 3 months, 6 months, and then each year after the operation. All patients visited our clinic at 3 months, 6 months, and then each year after the operation, and the symptoms, histopathology, and modified Rankin Scale score at the last follow-up were assessed.

### Surgical Technique

**Figure 2:**
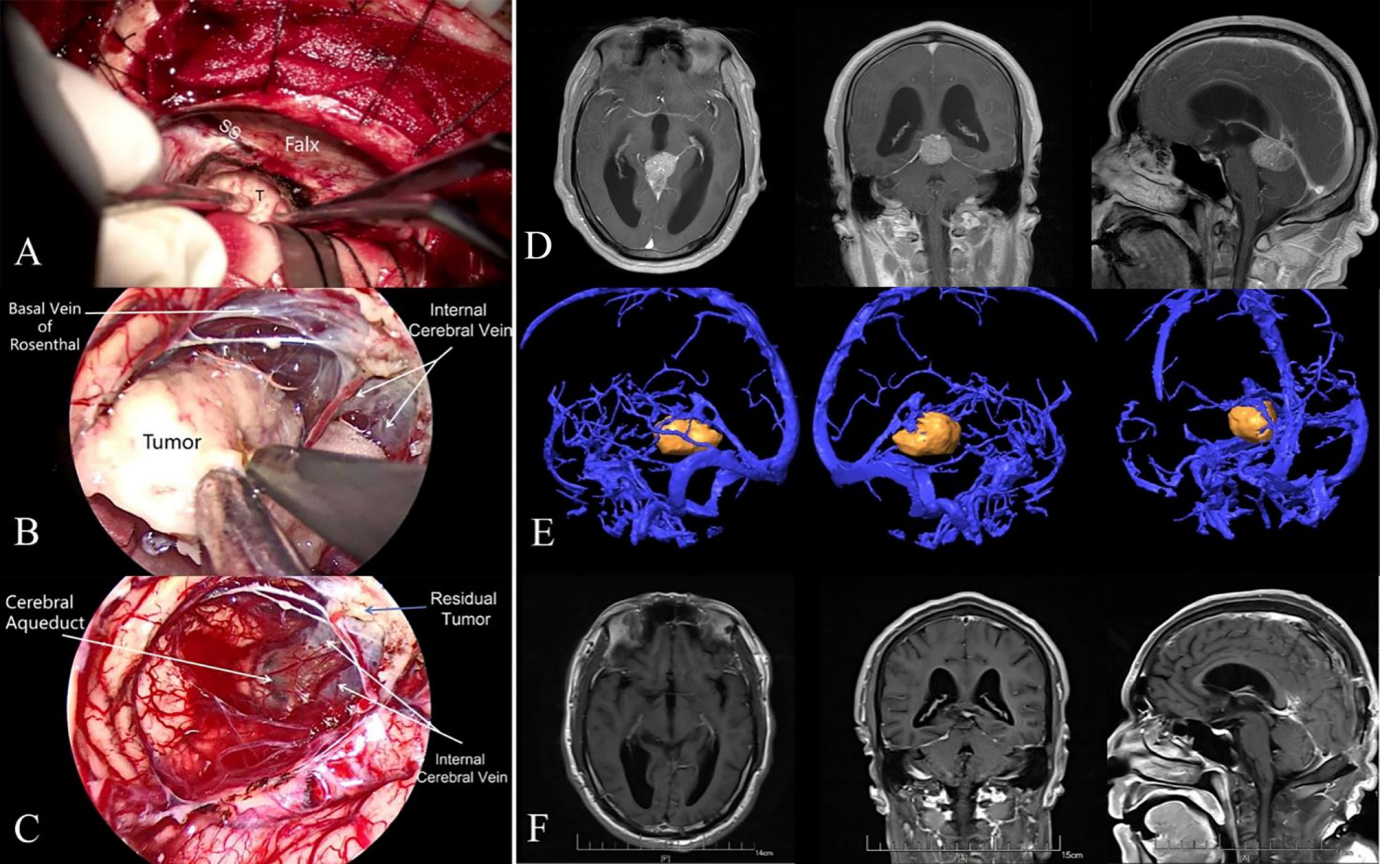
**(A)** Intraoperative microscopic view. Due to the occlusion of the falx cerebri, the microscopic view had a poor exposure of the internal cerebral vein and great cerebral vein. **(B)** Intraoperative endoscopic view. The endoscope increased the visualization of the deep venous system and allowed separating the tumor from the deep vein under direct vision. **(C)** Intraoperative endoscopic view. A small residual tumor was found adhering to the internal cerebral vein. **(D)** Preoperative contrast-enhanced T1-weighted MRI showed a pineal region meningioma in a 65-year-old man. **(E)** Multimodal fusion showed the meningioma lay underneath the deep venous complex. **(F)** Postoperative MRI confirmed a gross total resection at 3 months after surgery. *SS*, straight sinus; *T*, tumor.

The patient was placed in a 3/4 prone position with the head slightly rotated toward the contralateral side. This position allowed the occipital lobe to be retracted by gravity. A U-shaped skin incision was created, followed by an occipital craniotomy with a small bone window of approximately 3×5 cm. The bone window exposed the transverse and superior sagittal sinus margins. The dura mater was opened with a semicircular incision, based on the superior sagittal sinus. After gradual drainage of cerebrospinal fluid, the falx cerebri, straight sinus, and tentorium cerebelli were identified in turn under the microscope. A linear incision parallel to the straight sinus was made in the tentorium. Because most pineal region meningiomas originated near the falcotentorial junction receiving blood supply from the meningeal arteries along the tentorium and falx, we could eliminate the tumor blood supply early in most cases. However, the location of the tumor in relation to the deep veins could not be determined with the microscope at this time. We then inserted the endoscope under microscopic surveillance. After dissecting the arachnoid membrane, identified the vein of Galen and tributaries. The adhesion between the tumor and the deep veins was clearly exposed, which allowed the surgeon to separate the tumor from the veins under direct vision. At this stage, the endoscope could cross the falx, extend the contralateral operative field, and directly observe the rear of the third ventricle. In addition, once the suitable position was located, we fixed the endoscope with an endoscope holder, which allowed us to separate and dissect the tumor with both hands. In the final stage of surgery, the angled endoscope could look around the corner to detect any residual tumors. If the residual tumor was detected, it was resected with dedicated angled instruments under 30° visualization. Intraoperative photographs (**Figures 2A–C**) and MRI images (**Figures 2D–F**) of a patient with a pineal meningioma are shown in **Figure 2**.

## Results

**Figure 3:**
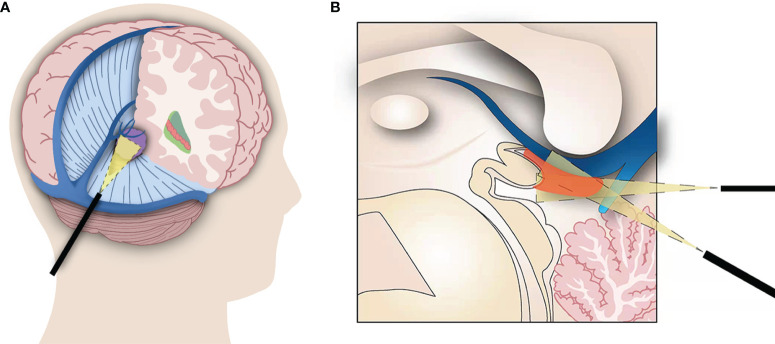
**(A)** Surgical approach of the combined microscopic and endoscopic surgery for pineal region meningiomas. The endoscope accesses the pineal region through the incision in the tentorium. **(B)** The endoscope improves visualization of the ventral aspect of the deep veins and the roof of the third ventricle, where tumors often remain. This combined technique also allows the tumor to be isolated from the veins under direct vision, reducing the risk of deep vein injury.

**Table 1** summarizes the clinical characteristics of the 12 patients with meningiomas in the pineal region. In this study, the mean follow-up period was 2.09 years (range, 8 months and 3.5 years). None of the patients died during the study period. Postoperative complications were observed in only one patient who presented with visual field defects, which resolved completely at 3 months after surgery. Five patients had preoperative hydrocephalus, and three of them received transient extraventricular drainage (< 10 days) after surgery; all patients were relieved of hydrocephalus. None of the patients required further ventriculoperitoneal (VP) shunts during follow-up. All deep veins were well protected, and no venous injury was observed during the operation. GTR was achieved in all patients. Total resection was believed to have been achieved in four patients, but the residual tumor was detected after endoscopic exploration and was completely resected using an endoscope. The residual tumor was often located at the ventral aspect of the deep veins or the roof of the third ventricle hidden by the corpus callosum (**Figure 2C**; **Figure 3**). Tumor recurrence was not detected during the follow-up period.

## Discussion

The pineal region is one of the most intricate anatomical regions in the human body. Resection of pineal region tumors is challenging, even for experienced neurosurgeons. Different from many pineal region malignant tumors, pineal region meningioma is a slow-growing benign tumor for which complete resection is the best treatment strategy. Therefore, despite surgical challenges, radical resection remains the primary treatment goal (15). In the present study, we treated 12 consecutive patients with meningiomas in the pineal region. All tumors were completely resected using the occipital-parietal transtentorial approach. We also found that the combined microscopic and endoscopic surgery is a promising technique.

The occipital transtentorial and supracerebellar infratentorial approaches are the most common approaches when accessing the pineal region. The supracerebellar infratentorial approach provides a posterior midline approach to the pineal region. The main advantage of this approach is that it utilizes the natural corridor between the superior cerebellar surface and tentorium without brain retraction. In addition, the pineal region is located underneath the major deep veins, which reduces the risk of damage to critical neurovascular structures (16, 17). However, this approach requires the sacrifice of midline bridging veins, leading to venous infarction and cerebellar edema. Furthermore, the supracerebellar infratentorial approach has poor visualization of supratentorial structures, and it is difficult to reach the lesions in the posterior floor of the third ventricle (8).

In contrast, the occipital transtentorial approach (OTA) provides the most extensive exposure of both the supratentorial and infratentorial compartments. It provides direct visualization of the deep venous structure and ipsilateral dorsal and lateral extension of the midbrain (15, 18). In this study, an OTA was used in all 12 patients, as we found that this approach has particular advantages for meningiomas in the pineal region. First, since most pineal region meningiomas derive their blood supply from the tentorium and falx, OTA allows for early devascularization of the feeding arteries along the tentorium and falx (18, 19). Second, meningiomas in the pineal region are slow growing, and there is a long duration between the onset of symptoms and diagnosis. Therefore, the tumor is usually relatively large at the time of detection and mostly grows in both the supratentorial and infratentorial compartments with lateral extension. The mean diameter of the tumors reported in this study was 3.8 cm, and the largest tumor diameter was 5.7 cm. Compared to the restricted visualization at the supratentorial structures in the supracerebellar infratentorial approach, OTA allows extensive exposure of both the supratentorial and infratentorial spaces and provides superior views of brainstem attachments. A previous study (12) suggested that the surgical approach should be selected according to the position of the tumor and the deep venous complex. The supracerebellar infratentorial approach is recommended for pineal region meningiomas displacing the deep venous complex superiorly. However, we believe that this positional relationship is not the sole determinant. When the meningioma lies underneath the deep venous complex, the OTA allows the surgeon to use either the interhemispheric or suboccipital supratentorial space; thus, the operator does not have to operate continuously among the deep veins.

However, the OTA also has the inherent limitations of restricted views around the neurovascular structures. This approach allows poor visualization of the ventral aspect of the vein of Galen, contralateral basal vein, and contralateral quadrigeminal region (**Figure 2A**). A transfalcine approach has been reported to improve exposure of the contralateral operative field through additional incisions above the sagittal sinus in the falx, but it is associated with significant risks (20). Meningiomas in the pineal region often compress the vital deep venous systems and develop collateral venous channels in the falx and tentorium. Additional incision of the falx could injure the collateral venous channels, resulting in venous infarction (19). In addition, the OTA is sometimes unable to expose the roof of the third ventricle, which is hidden by a prominent splenium (**Figure 3**), and sacrifice of the splenium may cause a disconnection syndrome.

In this study, we present a combined microscopic and endoscopic surgery for the resection of meningiomas in the pineal region using the OTA. This technique compensates for the shortcomings of the conventional microscopic OTA. First, the endoscope contributes to expanding the surgical field. The endoscope can cross the falx, extend the contralateral operative field, and increase the visualization of the contralateral internal cerebral vein and basal vein of Rosenthal. This allows the tumor to be isolated from the vein under direct vision, reducing the risk of deep vein injury (**Figure 2B**). In addition, through the tentorial incisura, the endoscope allows an extension of the microsurgical approach from the supratentorial region to the infratentorial region. Second, this combined microscopic and endoscopic surgery increases visualization of residual tumors. In the OTA, the ventral aspect of the vein of Galen and the roof of the third ventricle may not be adequately exposed using a microscope. The endoscope was invaluable in resolving the issue of hidden tumor remnants by allowing angled views around the deep venous structures and into the anterior third ventricle (21) (**Figure 2C**; **Figure 3**). In our series, total resection was believed to have been achieved in four patients, but the residual tumor was detected after endoscopic exploration and was completely resected using an endoscope.

However, insertion and movement of the endoscope may damage bridging veins and deep cerebral veins due to the lack of a posterior and lateral view. Some studies have indicated the importance of performing all endoscopic movements under microscopic surveillance (22, 23). However, when the microscope and endoscope are combined in the same setting, the surgeon has to switch between the microscope ocular view and the endoscope monitor view. This nonintegrated visual information can disrupt the flow of the operative procedure. Few studies have attempted to integrate endoscopes and microscopes. Here, we applied a picture-in-picture mode (**Figure 1**) in which microscopic and endoscopic images were merged on the same screen. With simultaneous microscope monitoring, the surgeon can safely place and move the endoscope and easily transition between the two modalities.

The pineal region is surrounded by critical neurovascular structures, such as the deep venous system, third ventricle, and midbrain. Despite the advancements in preoperative multimodal neuroimaging, intraoperative navigation, and microsurgical techniques, surgery for pineal region meningiomas remains a great challenge. The combined microscopic and endoscopic surgery using the OTA presented in this study provides an attractive treatment option for pineal region meningiomas. There were almost no blind spots in this approach. In our study, GTR was achieved in all 12 patients, and no recurrence occurred during the follow-up period. This ergonomic integration of the endoscope and microscope also reduced the risk of damage to critical neurovascular structures. No venous injury was observed during the operation, and there were no signs of brainstem injury after surgery. Five patients had preoperative hydrocephalus, which were relieved after surgery, and none of the patients required further VP shunts during the follow-up period. This study has some potential limitations. Because of the low incidence of pineal region meningiomas, this is a relatively small series of patients who underwent combined microscopic and endoscopic surgery, and we limited our analysis to a retrospective study. In the future, additional case–control studies should be conducted to validate our findings.

## Conclusion

The combined microscopic and endoscopic surgery for pineal region meningiomas eliminates microscope blind spots, thus compensating for the shortcomings of the traditional OTA. It is a promising technique for minimally invasive maximal resection of pineal region meningiomas.

## Data Availability Statement

The original contributions presented in the study are included in the article/supplementary material. Further inquiries can be directed to the corresponding authors.

## Ethics Statement

The studies involving human participants were reviewed and approved by Ethics committee, The First Affiliated Hospital of Soochow University. The patients/participants provided their written informed consent to participate in this study.

## Author Contributions

YD: Conceptualization, Methodology, Writing- Original draft preparation, Investigation. LS: Methodology, Writing- Original draft preparation, Investigation. YH: Formal analysis, Visualization. WZ: Investigation, Data Curation. LZ: Formal analysis, Investigation. ZY: Conceptualization, Methodology, Resources, Supervision, Project administration. JW: Conceptualization, Methodology, Resources, Writing - Review & Editing, Project administration. GC: Resources. All authors contributed to the article and approved the submitted version.

## Funding

This work was supported by Jiangsu Provincial Medical Key Talent grant (ZDRCA2016040) and grant (SYS2019045) from Suzhou Government, the grant from the National Natural Science Foundation of China (number 82171294), the National Natural Science Foundation for Youth of China (No. 81801137), and the Joint Funds of Jiangsu Province (yl2020lh05).

## Conflict of Interest

The authors declare that the research was conducted in the absence of any commercial or financial relationships that could be construed as a potential conflict of interest.

## Publisher’s Note

All claims expressed in this article are solely those of the authors and do not necessarily represent those of their affiliated organizations, or those of the publisher, the editors and the reviewers. Any product that may be evaluated in this article, or claim that may be made by its manufacturer, is not guaranteed or endorsed by the publisher.
